# Supplementary material for: Accurate Adapter Information Is Crucial for Reproducibility and Reusability in Small RNA Seq Studies
Source: Noncoding RNA. 2019 Oct 28;5(4):49. doi: 10.3390/ncrna5040049 (PMC6958438; doi:10.3390/ncrna5040049)
Supplement: Supplementary file 1 [file ncrna-05-00049-s001.pdf]

Supplementary Materials: Accurate adapter information is crucial for reproducible and reusable in small RNA seq studies

Xiangfu Zhong [1,*], Fatima Heinicke [1], Benedicte A. Lie [1] and Simon Rayner [1,2,*]

1. Materials and Methods

As a simple demonstration of the importance of using the correct adapter sequence for trimming, we retrieved two publicly available datasets [1,2] from the SRA, see data details in Supplementary Table S7. Dard-Dascot et al. [1] sequenced a library of six synthetic small RNAs (i.e., the sequences were known, and their length varied from 19 to 23 nt – see Supplementary Table S4 for details of selected dataset). The libraries were prepared by using both the NEBNext Small RNA Library Prep Kit and the CATS Small RNA-seq kit, as well as TruSeq, SMARTer and NEXTflex. The raw data was retrieved through accession number SRP128925 from the SRA. Gümürdü et al. [2] used NEBNext for library preparation; however, they specify a different adapter sequence in their publication. For reproducibility, we used the same version of cutadapt for trimming (v1.14) as specified in the original publication [1] and the Linux command *grep -c* was used for simple counting of reads that perfectly matched the known miRNAs. For adapter trimming of each sample, the adapter sequence was specified and the trimming and counting commands used for each sequence are given in Supplementary Table S8 and Supplementary Figure S1 B. The raw count information for each sequence is given in Supplementary Table S5 and Supplementary Table S6.

As the total library size prepared by different kits varied, for each query small RNA, we calculated the following percentage to measure the difference between trimming sets:

$$percentage = \frac{n_{i,j}}{N_{i,j}} \quad (1)$$

where $n_{i,j}$ is the number of perfectly matched reads for that small RNA_i in trimmed $data_j$, and $N_{i,j}$ is the number of reads containing the small RNA_i sequence in raw $data_j$.

Table S1. 3′ adapters for small RNA-seq kits for the Illumina platform.

3′Adapter Sequence	Vendor	Library Preparation Kit
5′-TGGAATTCTCGGGTGCCAAGG-3′	Illumina	TruSeq Small RNA Library Preparation Kit
5′-TGGAATTCTCGGGTGCCAAGG-3′	PerkinElmer	NEXTflex Small RNA Sequencing Kit *
5′-TGGAATTCTCGGGTGCCAAGG-3′	SeqMatic	TailorMix miRNA Sample Preparation Kit
5′-TGGAATTCTCGGGTGCCAAGG-3′	TriLink	CleanTag Small RNA Library Prep Kit
5′-AGATCGGAAGAGCACACGTCT-3′	New England Biolabs	NEBNext Multiplex Small RNA Library Prep Kit for Illumina
5′-AACTGTAGGCACCATCAAT-3′	Qiagen	QIAseq miRNA Library Kit
5′-TGGAATTCTCGGGTGCCAAGGAACTCCAGTCAC-3′	Lexogen	Small RNA-Seq Library Prep Kit
5′-AAAAAAAAAA-3′	Takara	SMARTer smRNA-Seq Kit
5′-GATCGGAAGAGCACACGTCTG-3′	Diagenode	CATS small RNA-seq Kit *

* Additional work required after adapter trimming to remove randomized nucleotides or poly(A).

Table S2. The number of SRA entries that mention library preparation kit in experimental description as of 27 May 2019.

**Kit name**	**Count**	**Percentage**
TruSeq	14111	33.91%
TrueSeq	335	0.81%
NEBNext	3590	8.63%
CATS	145	0.35%
NEXTflex	645	1.55%
TailorMix	89	0.21%
QIAseq	180	0.43%
CleanTag	280	0.67%
SMARTer	252	0.61%
Unspecific	21980	52.8 %
TOTAL ENTRIES	41607	

Table S3. Predicted adapter sequence from raw sequencing data using three tools, including Minion, DNApi and AdapterRemoval. Column 1: Kit Name; Column 2: SRA Run ID; Column 3: Adapter Sequence specified by kit manufacturer; Columns 4 to 6: Adapter sequence(s) predicted by Minion, DNApi and AdapterRemoval, respectively. In some cases more than one adapter sequence was predicted. In many cases, the adapter sequence is similar to, but not exactly equal to the adapter sequence specified by the kit manufacturer.

Kit	SRA Run	Adapter in kit manual	minion	DNApi	AdapterRemoval
NEBNext	SRR6464616	AGAT CGGA AGAG CACA CGTC T	<sequence-density> AGAT CGGA AGAG CACA CGTC TGAA CTCC AGTC ACCG ATGT ATCT CGTA TGC; <fanout-score> ACAG ATCG GAAG	AGAT CGGA AGAG	Adapter1[1]: AGAT CGGA AGAG CACA CGTC TGAA CTCC AGTC ACNN NNNN ATCT CGTA TGCC GTCT TCTG CTTG Adapter2[1]: AGAT CGGA AGAG CGTC GTGT AGGG AAAG AGTG TAGA TCTC GGTG GTCG CCGT ATCA TT
NEBNext	SRR6464623	AGAT CGGA AGAG CACA CGTC T	<sequence-density> AGAT CGGA AGAG CACA CGTC TGAA CTCC AGTC ACAT CACG ATCT CGTA TGC <fanout-score> GTCA GATC GGAA	AGAT CGGA AGAG	Adapter1[1]: AGAT CGGA AGAG CACA CGTC TGAA CTCC AGTC ACNN NNNN ATCT CGTA TGCC GTCT TCTG CTTG Adapter2[1]: AGAT CGGA AGAG CGTC GTGT AGGG AAAG AGTG TAGA TCTC GGTG GTCG CCGT ATCA TT
CATS	SRR6464674	GATC GGAA GAGC ACAC GTCT G	<sequence-density> GTCC TTCC AGAT GTTC GTTA G <fanout-score> ACGA TCCC CAGA TGGG ACAC	AAAA GATC GGA	Adapter1[1]: AGAT CGGA AGAG CACA CGTC TGAA CTCC AGTC ACNN NNNN ATCT CGTA TGCC GTCT TCTG CTTG Adapter2[1]: AGAT CGGA AGAG CGTC GTGT AGGG AAAG AGTG TAGA TCTC GGTG GTCG CCGT ATCA TT
CATS	SRR6464673	GATC GGAA GAGC ACAC GTCT G	<sequence-density> GTCC TTCC AGAT GTTC GTTA G <fanout-score> ACGA TCGG GAGA TGGG ACAC CTTG TGAC T	AAAA GATC GGA	Adapter1[1]: AGAT CGGA AGAG CACA CGTC TGAA CTCC AGTC ACNN NNNN ATCT CGTA TGCC GTCT TCTG CTTG Adapter2[1]: AGAT CGGA AGAG CGTC GTGT AGGG AAAG AGTG TAGA TCTC GGTG GTCG CCGT ATCA TT

Continued on next page

Table S3 – *Continued from previous page*

Kit	SRA Run	Adapter in kit manual	minion	DNApi	AdapterRemoval
NEXTflex	SRR6464672	TGGA ATTC TCGG GTGC CAAG G	<sequence-density> TGGA ATTC TCGG GTGC CAAG GAAC TCCA GTCA CCAG ATCA TCTC GTAT GCC <fanout-score> GAAT TCTC GGGG GCCA AGGA ACT	TGGA ATTC TCGG	Adapter1[1]: AGAT CGGA AGAG CACA CGTC TGAA CTCC AGTC ACNN NNNN ATCT CGTA TGCC GTCT TCTG CTTG Adapter2[1]: AGAT CGGA AGAG CGTC GTGT AGGG AAAG AGTG TAGA TCTC GGTG GTCG CCGT ATCA TT
NEXTflex	SRR6464671	TGGA ATTC TCGG GTGC CAAG G	<sequence-density> TGGA ATTC TCGG GTGC CAAG GAAC TCCA GTCA CCCA ACAA TCTC GTAT GC <fanout-score> GTCT GGAA TTCT	TGGA ATTC TCGG	Adapter1[1]: AGAT CGGA AGAG CACA CGTC TGAA CTCC AGTC ACNN NNNN ATCT CGTA TGCC GTCT TCTG CTTG Adapter2[1]: AGAT CGGA AGAG CGTC GTGT AGGG AAAG AGTG TAGA TCTC GGTG GTCG CCGT ATCA TT
TruSeq	SRR6464662	TGGA ATTC TCGG GTGC CAAG G	<sequence-density> TGGA ATTC TCGG GTGC CAAG GAAC TCCA GTCA CCGG AATA TCTC GTAT GC <fanout-score> GTCT GGAA TTCT	TGGA ATTC TCGG	Adapter1[1]: AGAT CGGA AGAG CACA CGTC TGAA CTCC AGTC ACNN NNNN ATCT CGTA TGCC GTCT TCTG CTTG Adapter2[1]: AGAT CGGA AGAG CGTC GTGT AGGG AAAG AGTG TAGA TCTC GGTG GTCG CCGT ATCA TT
TruSeq	SRR6464661	TGGA ATTC TCGG GTGC CAAG G	<sequence-density> TGGA ATTC TCGG GTGC CAAG GAAC TCCA GTCA CCCA ACAA TCTC GTAT GC <fanout-score> GTCT GGAA TTCT	TGGA ATTC TCGG	Adapter1[1]: AGAT CGGA AGAG CACA CGTC TGAA CTCC AGTC ACNN NNNN ATCT CGTA TGCC GTCT TCTG CTTG Adapter2[1]: AGAT CGGA AGAG CGTC GTGT AGGG AAAG AGTG TAGA TCTC GGTG GTCG CCGT ATCA TT

Continued on next page

Table S3 – *Continued from previous page*

**Kit**	**SRA Run**	**Adapter in kit manual**	**minion**	**DNApi**	**AdapterRemoval**
SMARTer	SRR6464721	AAAA AAAA AA	<sequence-density> AAAA AAAA AAGA TCGG AAGA GCAC ACGT CTGA ACTC CAGT CACT CCGG AGAA T <fanout-score> AAAG ATCG GAAC AGCA CACG TCT	AAAA GATC GGAA G	Adapter1[1]: AGAT CGGA AGAG CACA CGTC TGAA CTCC AGTC ACNN NNNN ATCT CGTA TGCC GTCT TCTG CTTG Adapter2[1]: AGAT CGGA AGAG CGTC GTGT AGGG AAAG AGTG TAGA TCTC GGTG GTCG CCGT ATCA TT
SMARTer	SRR6464720	AAAA AAAA AA	<sequence-density> AAAA AAAA AAGA TCGG AAGA GCAC ACGT CTGA ACTC CAGT CACT CCGC GAAA T <fanout-score> AAAG ATCG GAAC AGCA CACG TCT	AAAA GATC GGAA GAGC AC	Adapter1[1]: AGAT CGGA AGAG CACA CGTC TGAA CTCC AGTC ACNN NNNN ATCT CGTA TGCC GTCT TCTG CTTG Adapter2[1]: AGAT CGGA AGAG CGTC GTGT AGGG AAAG AGTG TAGA TCTC GGTG GTCG CCGT ATCA TT

Table S4. Sequences for the six synthetic oligonucleotides from [1] shown in Figure 1 and Table S5.

Oligo Name	Oligonucleotides Sequence
RNA1	TGTGTTTGTGGAGATATGACATC
RNA2	AGATGGGACACCTTGTGACTAA
RNA3	GTCCTTCCAGATGTTCGTTAG
RNA4	TCCCTAAGGACCCACACGTTA
RNA5	TACAAATGTAGTGTATCCTT
RNA6	CCCATAATCCTAGTTATGG

Table S5. Un-transformed count table for samples in dataset [1] for raw data and using the various adapter sequences as specified in Figure 1.

FASTQ	Trimmed Read Set	RNA1	RNA2	RNA3	RNA4	RNA5	RNA6
SRR6464616	RAW	261372	21147	723	261	5953	21
SRR6464616	NEBNext_trim01	260260	20159	139	101	5512	1
SRR6464616	NEBNext_trim02	381	1	0	0	9	0
SRR6464616	NEBNext_trim03	224	244	0	5	5	0
SRR6464623	RAW	214535	19227	975	262	7843	21
SRR6464623	NEBNext_trim01	213482	18106	170	81	7237	0
SRR6464623	NEBNext_trim02	421	5	2	1	8	0
SRR6464623	NEBNext_trim03	43	239	0	6	0	0
SRR6464673	RAW	4121	13607	5574	4500	1427	209
SRR6464673	CATS_trim01	0	0	0	0	0	0
SRR6464673	CATS_trim02	0	0	0	0	0	0
SRR6464673	CATS_trim03	3630	5	4993	0	1226	126
SRR6464673	CATS_trim04	3630	5	4993	0	1226	126
SRR6464673	CATS_trim05	500	5	184	0	1101	8
SRR6464673	CATS_trim06	521	5	184	0	1096	18
SRR6464674	RAW	5736	16502	7915	6689	2749	253
SRR6464674	CATS_trim01	0	0	0	0	0	0
SRR6464674	CATS_trim02	0	0	0	0	0	0
SRR6464674	CATS_trim03	5018	7	7161	0	2406	146
SRR6464674	CATS_trim04	5018	7	7161	0	2406	146
SRR6464674	CATS_trim05	780	7	241	0	2181	17
SRR6464674	CATS_trim06	804	7	241	0	2177	23

(A)

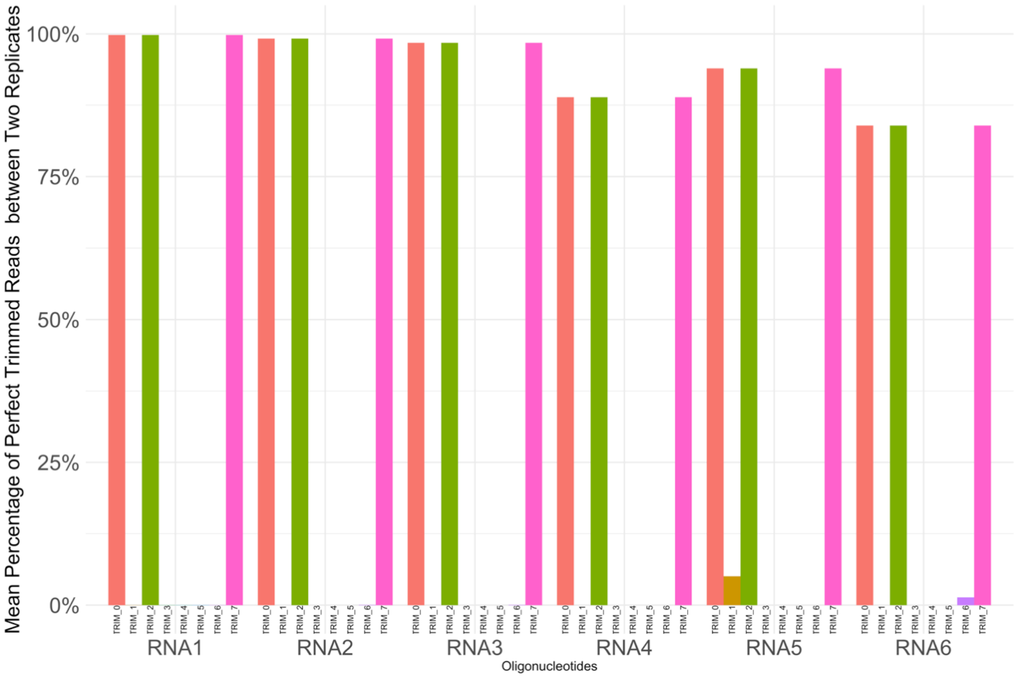

(B)

```
TruSeq TRIM_0    TGGAATTCTCGGGTGCCAAGG
TruSeq TRIM_1     GGAATTCTCGGGTGCCAAGG
TruSeq TRIM_2    TGGAATTCTCGGGTGCCAAG
TruSeq TRIM_3   ATGGAATTCTCGGGTGCCAAGG
TruSeq TRIM_4   GTGGAATTCTCGGGTGCCAAGG
TruSeq TRIM_5   TTGGAATTCTCGGGTGCCAAGG
TruSeq TRIM_6   CTGGAATTCTCGGGTGCCAAGG
TruSeq TRIM_7    TGGAATTCTCGG
```

Figure S1. Use of incorrect adapter sequence or trimming protocol can lead to miscounting of reads mapping to features. Sequencing data which used TruSeq library protocol were retrieved from the SRA, submitted by [1]. (A) The mean percentage of perfect trimmed reads in TruSeq prepared dataset varies according to the eight different adapter sequences used for trimming varies according to the different but higher similar adapter sequences. For TRIM_0, TRIM_2 and TRIM_7, more than 75% of raw reads are perfectly trimmed across all six synthetic RNA oligonucleotides and two replicates. (B) The sequence alignment of adapter sequence used in (A). TruSeq TRIM_0: the correct TruSeq adapter sequence, marked in red. Sequence for oligonucleotides RNA1-6 are same as Figure 1 and listed in *Supplementary Table S7*. TruSeq TRIM_0: the correct TruSeq adapter sequence, marked in red; TruSeq TRIM_1: first nucleotide removed at 5′ end from the correct sequence; TruSeq TRIM_2: last nucleotide removed at the 3′ end; TruSeq TRIM_3: additional A added at the 5′ end; TruSeq TRIM_4: additional G added at the 5′ end; TruSeq TRIM_5: additional T added at the 5′ end; TruSeq TRIM_6: additional C added at the 5′ end; TruSeq TRIM_7: the adapter sequence identified from raw data by *DNApi*.

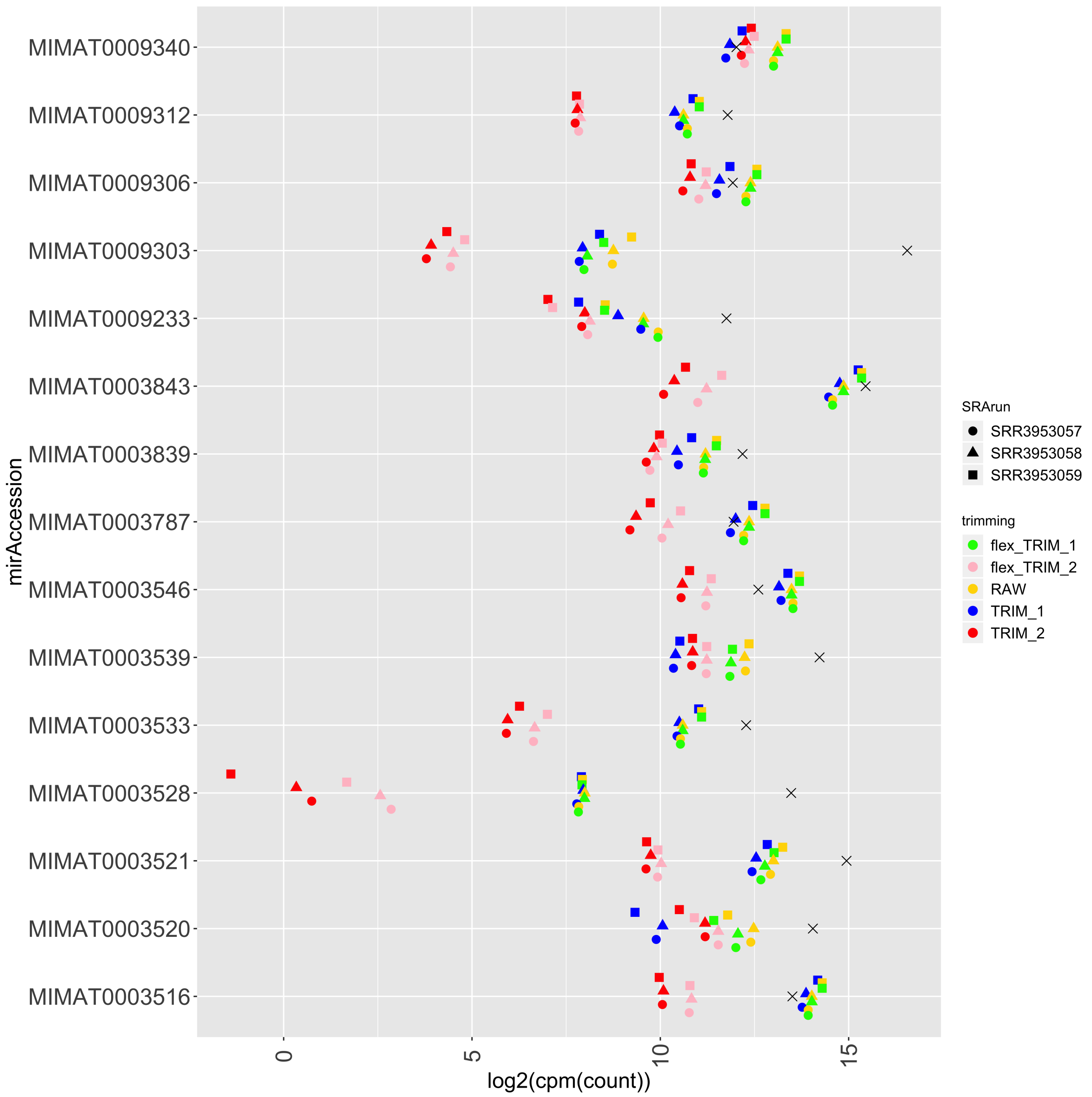

Figure S2. Trimming analysis results for dataset from [2]. X-axis shows the log 2 of the counts per million (CPM). Y-axis: miRNAs listed in Table 1 in the original publication. Coloured dots correspond to count data generated in this analysis. Black crosses correspond to count data from the original publication. Shape corresponds to SRA run, colour corresponds to trimming method. Legend is shown underneath the plot. Trimming options: *RAW*: "grep" and "count" was used to find reads containing the query miRNA sequence; *TRIM_1* "grep" and "count" was used to find all NEBNext adapter sequence trimmed reads that **perfectly matched** the query miRNA sequence; *TRIM_2*: "grep" and "count" was used to find all mutated (with an additional A at the 5′ end) NEBNext adapter sequence reads that **perfectly matched** the query miRNA sequence; *flex_TRIM_1*: as *TRIM_1*, but counting all reads **containing** the query miRNA sequence; *flex_TRIM_2*: as *TRIM_2*, but counting all reads **containing** the query miRNA sequence. In most cases, the black dots are above coloured dots, implying that direct comparison with their results could not achieved. However, the distinct difference between *TRIM_1* (blue) and *TRIM_2* (red) indicate that adapter trimming result is sensitive to a single nucleotide change at the 5′ end that can be introduced by misspecification of the adapter sequence.

Table S6. Trimming results for top 15 most abundant miRNAs in [2]

Accession	Sequence	SRA run	type	count
MIMAT0003520	TACAGTACTGTGATAACTGAA	SRR3953057	RAW	300207
MIMAT0003520	TACAGTACTGTGATAACTGAA	SRR3953057	TRIM_1	52682
MIMAT0003520	TACAGTACTGTGATAACTGAA	SRR3953057	TRIM_2	129684
MIMAT0003520	TACAGTACTGTGATAACTGAA	SRR3953057	flex_TRIM_1	228037
MIMAT0003520	TACAGTACTGTGATAACTGAA	SRR3953057	flex_TRIM_2	164910
MIMAT0003520	TACAGTACTGTGATAACTGAA	SRR3953058	RAW	316465
MIMAT0003520	TACAGTACTGTGATAACTGAA	SRR3953058	TRIM_1	59252
MIMAT0003520	TACAGTACTGTGATAACTGAA	SRR3953058	TRIM_2	129400
MIMAT0003520	TACAGTACTGTGATAACTGAA	SRR3953058	flex_TRIM_1	237092
MIMAT0003520	TACAGTACTGTGATAACTGAA	SRR3953058	flex_TRIM_2	165013
MIMAT0003520	TACAGTACTGTGATAACTGAA	SRR3953059	RAW	196160
MIMAT0003520	TACAGTACTGTGATAACTGAA	SRR3953059	TRIM_1	35651
MIMAT0003520	TACAGTACTGTGATAACTGAA	SRR3953059	TRIM_2	80593
MIMAT0003520	TACAGTACTGTGATAACTGAA	SRR3953059	flex_TRIM_1	151815
MIMAT0003520	TACAGTACTGTGATAACTGAA	SRR3953059	flex_TRIM_2	106318
MIMAT0003521	AGCAGCATTGTACAGGGCTATGA	SRR3953057	RAW	431423
MIMAT0003521	AGCAGCATTGTACAGGGCTATGA	SRR3953057	TRIM_1	307374
MIMAT0003521	AGCAGCATTGTACAGGGCTATGA	SRR3953057	TRIM_2	43656
MIMAT0003521	AGCAGCATTGTACAGGGCTATGA	SRR3953057	flex_TRIM_1	361215
MIMAT0003521	AGCAGCATTGTACAGGGCTATGA	SRR3953057	flex_TRIM_2	54033
MIMAT0003521	AGCAGCATTGTACAGGGCTATGA	SRR3953058	RAW	455053
MIMAT0003521	AGCAGCATTGTACAGGGCTATGA	SRR3953058	TRIM_1	331253
MIMAT0003521	AGCAGCATTGTACAGGGCTATGA	SRR3953058	TRIM_2	47616
MIMAT0003521	AGCAGCATTGTACAGGGCTATGA	SRR3953058	flex_TRIM_1	388782
MIMAT0003521	AGCAGCATTGTACAGGGCTATGA	SRR3953058	flex_TRIM_2	57703
MIMAT0003521	AGCAGCATTGTACAGGGCTATGA	SRR3953059	RAW	539943
MIMAT0003521	AGCAGCATTGTACAGGGCTATGA	SRR3953059	TRIM_1	406512
MIMAT0003521	AGCAGCATTGTACAGGGCTATGA	SRR3953059	TRIM_2	44146
MIMAT0003521	AGCAGCATTGTACAGGGCTATGA	SRR3953059	flex_TRIM_1	460659
MIMAT0003521	AGCAGCATTGTACAGGGCTATGA	SRR3953059	flex_TRIM_2	54367
MIMAT0003839	TACCCTGTAGAACCGAATTTGTG	SRR3953057	RAW	126131
MIMAT0003839	TACCCTGTAGAACCGAATTTGTG	SRR3953057	TRIM_1	79221
MIMAT0003839	TACCCTGTAGAACCGAATTTGTG	SRR3953057	TRIM_2	43869
MIMAT0003839	TACCCTGTAGAACCGAATTTGTG	SRR3953057	flex_TRIM_1	125426
MIMAT0003839	TACCCTGTAGAACCGAATTTGTG	SRR3953057	flex_TRIM_2	46893
MIMAT0003839	TACCCTGTAGAACCGAATTTGTG	SRR3953058	RAW	130574
MIMAT0003839	TACCCTGTAGAACCGAATTTGTG	SRR3953058	TRIM_1	77208
MIMAT0003839	TACCCTGTAGAACCGAATTTGTG	SRR3953058	TRIM_2	50411
MIMAT0003839	TACCCTGTAGAACCGAATTTGTG	SRR3953058	flex_TRIM_1	129680
MIMAT0003839	TACCCTGTAGAACCGAATTTGTG	SRR3953058	flex_TRIM_2	53066
MIMAT0003839	TACCCTGTAGAACCGAATTTGTG	SRR3953059	RAW	160119
MIMAT0003839	TACCCTGTAGAACCGAATTTGTG	SRR3953059	TRIM_1	101131
MIMAT0003839	TACCCTGTAGAACCGAATTTGTG	SRR3953059	TRIM_2	56002
MIMAT0003839	TACCCTGTAGAACCGAATTTGTG	SRR3953059	flex_TRIM_1	159191
MIMAT0003839	TACCCTGTAGAACCGAATTTGTG	SRR3953059	flex_TRIM_2	58887
MIMAT0003539	TCCCTGAGACCCTAACTTGTGA	SRR3953057	RAW	272134
MIMAT0003539	TCCCTGAGACCCTAACTTGTGA	SRR3953057	TRIM_1	72372

Continued on next page

Table S6 – *Continued from previous page*

Accession	Sequence	SRA run	type	count
MIMAT0003539	TCCCTGAGACCCTAACTTGTGA	SRR3953057	TRIM_2	101107
MIMAT0003539	TCCCTGAGACCCTAACTTGTGA	SRR3953057	flex_TRIM_1	204384
MIMAT0003539	TCCCTGAGACCCTAACTTGTGA	SRR3953057	flex_TRIM_2	132317
MIMAT0003539	TCCCTGAGACCCTAACTTGTGA	SRR3953058	RAW	267992
MIMAT0003539	TCCCTGAGACCCTAACTTGTGA	SRR3953058	TRIM_1	75142
MIMAT0003539	TCCCTGAGACCCTAACTTGTGA	SRR3953058	TRIM_2	103097
MIMAT0003539	TCCCTGAGACCCTAACTTGTGA	SRR3953058	flex_TRIM_1	208384
MIMAT0003539	TCCCTGAGACCCTAACTTGTGA	SRR3953058	flex_TRIM_2	133561
MIMAT0003539	TCCCTGAGACCCTAACTTGTGA	SRR3953059	RAW	290586
MIMAT0003539	TCCCTGAGACCCTAACTTGTGA	SRR3953059	TRIM_1	81350
MIMAT0003539	TCCCTGAGACCCTAACTTGTGA	SRR3953059	TRIM_2	102712
MIMAT0003539	TCCCTGAGACCCTAACTTGTGA	SRR3953059	flex_TRIM_1	214334
MIMAT0003539	TCCCTGAGACCCTAACTTGTGA	SRR3953059	flex_TRIM_2	133431
MIMAT0003787	TCGGATCCGTCTGAGCTTGGCT	SRR3953057	RAW	263225
MIMAT0003787	TCGGATCCGTCTGAGCTTGGCT	SRR3953057	TRIM_1	206437
MIMAT0003787	TCGGATCCGTCTGAGCTTGGCT	SRR3953057	TRIM_2	32466
MIMAT0003787	TCGGATCCGTCTGAGCTTGGCT	SRR3953057	flex_TRIM_1	263194
MIMAT0003787	TCGGATCCGTCTGAGCTTGGCT	SRR3953057	flex_TRIM_2	58544
MIMAT0003787	TCGGATCCGTCTGAGCTTGGCT	SRR3953058	RAW	290963
MIMAT0003787	TCGGATCCGTCTGAGCTTGGCT	SRR3953058	TRIM_1	227280
MIMAT0003787	TCGGATCCGTCTGAGCTTGGCT	SRR3953058	TRIM_2	36351
MIMAT0003787	TCGGATCCGTCTGAGCTTGGCT	SRR3953058	flex_TRIM_1	290915
MIMAT0003787	TCGGATCCGTCTGAGCTTGGCT	SRR3953058	flex_TRIM_2	65550
MIMAT0003787	TCGGATCCGTCTGAGCTTGGCT	SRR3953059	RAW	389838
MIMAT0003787	TCGGATCCGTCTGAGCTTGGCT	SRR3953059	TRIM_1	311097
MIMAT0003787	TCGGATCCGTCTGAGCTTGGCT	SRR3953059	TRIM_2	47221
MIMAT0003787	TCGGATCCGTCTGAGCTTGGCT	SRR3953059	flex_TRIM_1	389796
MIMAT0003787	TCGGATCCGTCTGAGCTTGGCT	SRR3953059	flex_TRIM_2	82401
MIMAT0009233	TGAGATGAAGCACTGTAGCTCG	SRR3953057	RAW	54663
MIMAT0009233	TGAGATGAAGCACTGTAGCTCG	SRR3953057	TRIM_1	39650
MIMAT0009233	TGAGATGAAGCACTGTAGCTCG	SRR3953057	TRIM_2	13374
MIMAT0009233	TGAGATGAAGCACTGTAGCTCG	SRR3953057	flex_TRIM_1	54380
MIMAT0009233	TGAGATGAAGCACTGTAGCTCG	SRR3953057	flex_TRIM_2	14928
MIMAT0009233	TGAGATGAAGCACTGTAGCTCG	SRR3953058	RAW	41830
MIMAT0009233	TGAGATGAAGCACTGTAGCTCG	SRR3953058	TRIM_1	26118
MIMAT0009233	TGAGATGAAGCACTGTAGCTCG	SRR3953058	TRIM_2	14157
MIMAT0009233	TGAGATGAAGCACTGTAGCTCG	SRR3953058	flex_TRIM_1	41520
MIMAT0009233	TGAGATGAAGCACTGTAGCTCG	SRR3953058	flex_TRIM_2	15527
MIMAT0009233	TGAGATGAAGCACTGTAGCTCG	SRR3953059	RAW	20612
MIMAT0009233	TGAGATGAAGCACTGTAGCTCG	SRR3953059	TRIM_1	12630
MIMAT0009233	TGAGATGAAGCACTGTAGCTCG	SRR3953059	TRIM_2	7178
MIMAT0009233	TGAGATGAAGCACTGTAGCTCG	SRR3953059	flex_TRIM_1	20366
MIMAT0009233	TGAGATGAAGCACTGTAGCTCG	SRR3953059	flex_TRIM_2	7822
MIMAT0003528	TAGCTTATCAGACTGATGTTGACT	SRR3953057	RAW	12660
MIMAT0003528	TAGCTTATCAGACTGATGTTGACT	SRR3953057	TRIM_1	12209
MIMAT0003528	TAGCTTATCAGACTGATGTTGACT	SRR3953057	TRIM_2	93
MIMAT0003528	TAGCTTATCAGACTGATGTTGACT	SRR3953057	flex_TRIM_1	12583

Continued on next page

Table S6 – *Continued from previous page*

Accession	Sequence	SRA run	type	count
MIMAT0003528	TAGCTTATCAGACTGATGTTGACT	SRR3953057	flex_TRIM_2	401
MIMAT0003528	TAGCTTATCAGACTGATGTTGACT	SRR3953058	RAW	14137
MIMAT0003528	TAGCTTATCAGACTGATGTTGACT	SRR3953058	TRIM_1	13765
MIMAT0003528	TAGCTTATCAGACTGATGTTGACT	SRR3953058	TRIM_2	70
MIMAT0003528	TAGCTTATCAGACTGATGTTGACT	SRR3953058	flex_TRIM_1	14039
MIMAT0003528	TAGCTTATCAGACTGATGTTGACT	SRR3953058	flex_TRIM_2	328
MIMAT0003528	TAGCTTATCAGACTGATGTTGACT	SRR3953059	RAW	13533
MIMAT0003528	TAGCTTATCAGACTGATGTTGACT	SRR3953059	TRIM_1	13300
MIMAT0003528	TAGCTTATCAGACTGATGTTGACT	SRR3953059	TRIM_2	21
MIMAT0003528	TAGCTTATCAGACTGATGTTGACT	SRR3953059	flex_TRIM_1	13435
MIMAT0003528	TAGCTTATCAGACTGATGTTGACT	SRR3953059	flex_TRIM_2	177
MIMAT0003516	TTCAAGTAATCCAGGATAGGCT	SRR3953057	RAW	863437
MIMAT0003516	TTCAAGTAATCCAGGATAGGCT	SRR3953057	TRIM_1	773049
MIMAT0003516	TTCAAGTAATCCAGGATAGGCT	SRR3953057	TRIM_2	59043
MIMAT0003516	TTCAAGTAATCCAGGATAGGCT	SRR3953057	flex_TRIM_1	863398
MIMAT0003516	TTCAAGTAATCCAGGATAGGCT	SRR3953057	flex_TRIM_2	96709
MIMAT0003516	TTCAAGTAATCCAGGATAGGCT	SRR3953058	RAW	923027
MIMAT0003516	TTCAAGTAATCCAGGATAGGCT	SRR3953058	TRIM_1	829847
MIMAT0003516	TTCAAGTAATCCAGGATAGGCT	SRR3953058	TRIM_2	60192
MIMAT0003516	TTCAAGTAATCCAGGATAGGCT	SRR3953058	flex_TRIM_1	922985
MIMAT0003516	TTCAAGTAATCCAGGATAGGCT	SRR3953058	flex_TRIM_2	100897
MIMAT0003516	TTCAAGTAATCCAGGATAGGCT	SRR3953059	RAW	1116547
MIMAT0003516	TTCAAGTAATCCAGGATAGGCT	SRR3953059	TRIM_1	1029020
MIMAT0003516	TTCAAGTAATCCAGGATAGGCT	SRR3953059	TRIM_2	55682
MIMAT0003516	TTCAAGTAATCCAGGATAGGCT	SRR3953059	flex_TRIM_1	1116498
MIMAT0003516	TTCAAGTAATCCAGGATAGGCT	SRR3953059	flex_TRIM_2	97986
MIMAT0003546	TTCACAGTGGCTAAGTTCTGC	SRR3953057	RAW	652277
MIMAT0003546	TTCACAGTGGCTAAGTTCTGC	SRR3953057	TRIM_1	523726
MIMAT0003546	TTCACAGTGGCTAAGTTCTGC	SRR3953057	TRIM_2	83238
MIMAT0003546	TTCACAGTGGCTAAGTTCTGC	SRR3953057	flex_TRIM_1	652231
MIMAT0003546	TTCACAGTGGCTAAGTTCTGC	SRR3953057	flex_TRIM_2	131203
MIMAT0003546	TTCACAGTGGCTAAGTTCTGC	SRR3953058	RAW	635793
MIMAT0003546	TTCACAGTGGCTAAGTTCTGC	SRR3953058	TRIM_1	504600
MIMAT0003546	TTCACAGTGGCTAAGTTCTGC	SRR3953058	TRIM_2	85267
MIMAT0003546	TTCACAGTGGCTAAGTTCTGC	SRR3953058	flex_TRIM_1	635745
MIMAT0003546	TTCACAGTGGCTAAGTTCTGC	SRR3953058	flex_TRIM_2	134088
MIMAT0003546	TTCACAGTGGCTAAGTTCTGC	SRR3953059	RAW	735477
MIMAT0003546	TTCACAGTGGCTAAGTTCTGC	SRR3953059	TRIM_1	594271
MIMAT0003546	TTCACAGTGGCTAAGTTCTGC	SRR3953059	TRIM_2	97342
MIMAT0003546	TTCACAGTGGCTAAGTTCTGC	SRR3953059	flex_TRIM_1	735444
MIMAT0003546	TTCACAGTGGCTAAGTTCTGC	SRR3953059	flex_TRIM_2	145033
MIMAT0003533	TGTAAACATCCCCGACTGGAAGCT	SRR3953057	RAW	82257
MIMAT0003533	TGTAAACATCCCCGACTGGAAGCT	SRR3953057	TRIM_1	77263
MIMAT0003533	TGTAAACATCCCCGACTGGAAGCT	SRR3953057	TRIM_2	3342
MIMAT0003533	TGTAAACATCCCCGACTGGAAGCT	SRR3953057	flex_TRIM_1	82242
MIMAT0003533	TGTAAACATCCCCGACTGGAAGCT	SRR3953057	flex_TRIM_2	5505
MIMAT0003533	TGTAAACATCCCCGACTGGAAGCT	SRR3953058	RAW	85714

Continued on next page

Table S6 – *Continued from previous page*

Accession	Sequence	SRA run	type	count
MIMAT0003533	TGTAAACATCCCCGACTGGAAGCT	SRR3953058	TRIM_1	80601
MIMAT0003533	TGTAAACATCCCCGACTGGAAGCT	SRR3953058	TRIM_2	3420
MIMAT0003533	TGTAAACATCCCCGACTGGAAGCT	SRR3953058	flex_TRIM_1	85693
MIMAT0003533	TGTAAACATCCCCGACTGGAAGCT	SRR3953058	flex_TRIM_2	5623
MIMAT0003533	TGTAAACATCCCCGACTGGAAGCT	SRR3953059	RAW	121424
MIMAT0003533	TGTAAACATCCCCGACTGGAAGCT	SRR3953059	TRIM_1	115040
MIMAT0003533	TGTAAACATCCCCGACTGGAAGCT	SRR3953059	TRIM_2	4257
MIMAT0003533	TGTAAACATCCCCGACTGGAAGCT	SRR3953059	flex_TRIM_1	121376
MIMAT0003533	TGTAAACATCCCCGACTGGAAGCT	SRR3953059	flex_TRIM_2	7110
MIMAT0009303	TTTTGTTCGTTCGGCTCGCGTGA	SRR3953057	RAW	23525
MIMAT0009303	TTTTGTTCGTTCGGCTCGCGTGA	SRR3953057	TRIM_1	12781
MIMAT0009303	TTTTGTTCGTTCGGCTCGCGTGA	SRR3953057	TRIM_2	767
MIMAT0009303	TTTTGTTCGTTCGGCTCGCGTGA	SRR3953057	flex_TRIM_1	13939
MIMAT0009303	TTTTGTTCGTTCGGCTCGCGTGA	SRR3953057	flex_TRIM_2	1193
MIMAT0009303	TTTTGTTCGTTCGGCTCGCGTGA	SRR3953058	RAW	23952
MIMAT0009303	TTTTGTTCGTTCGGCTCGCGTGA	SRR3953058	TRIM_1	13594
MIMAT0009303	TTTTGTTCGTTCGGCTCGCGTGA	SRR3953058	TRIM_2	837
MIMAT0009303	TTTTGTTCGTTCGGCTCGCGTGA	SRR3953058	flex_TRIM_1	14808
MIMAT0009303	TTTTGTTCGTTCGGCTCGCGTGA	SRR3953058	flex_TRIM_2	1258
MIMAT0009303	TTTTGTTCGTTCGGCTCGCGTGA	SRR3953059	RAW	33483
MIMAT0009303	TTTTGTTCGTTCGGCTCGCGTGA	SRR3953059	TRIM_1	18570
MIMAT0009303	TTTTGTTCGTTCGGCTCGCGTGA	SRR3953059	TRIM_2	1117
MIMAT0009303	TTTTGTTCGTTCGGCTCGCGTGA	SRR3953059	flex_TRIM_1	20049
MIMAT0009303	TTTTGTTCGTTCGGCTCGCGTGA	SRR3953059	flex_TRIM_2	1552
MIMAT0009306	TGGTAGACTATGGAACGTAGG	SRR3953057	RAW	274449
MIMAT0009306	TGGTAGACTATGGAACGTAGG	SRR3953057	TRIM_1	159699
MIMAT0009306	TGGTAGACTATGGAACGTAGG	SRR3953057	TRIM_2	85999
MIMAT0009306	TGGTAGACTATGGAACGTAGG	SRR3953057	flex_TRIM_1	274382
MIMAT0009306	TGGTAGACTATGGAACGTAGG	SRR3953057	flex_TRIM_2	115348
MIMAT0009306	TGGTAGACTATGGAACGTAGG	SRR3953058	RAW	298674
MIMAT0009306	TGGTAGACTATGGAACGTAGG	SRR3953058	TRIM_1	168685
MIMAT0009306	TGGTAGACTATGGAACGTAGG	SRR3953058	TRIM_2	98152
MIMAT0009306	TGGTAGACTATGGAACGTAGG	SRR3953058	flex_TRIM_1	298577
MIMAT0009306	TGGTAGACTATGGAACGTAGG	SRR3953058	flex_TRIM_2	130630
MIMAT0009306	TGGTAGACTATGGAACGTAGG	SRR3953059	RAW	335852
MIMAT0009306	TGGTAGACTATGGAACGTAGG	SRR3953059	TRIM_1	204629
MIMAT0009306	TGGTAGACTATGGAACGTAGG	SRR3953059	TRIM_2	100214
MIMAT0009306	TGGTAGACTATGGAACGTAGG	SRR3953059	flex_TRIM_1	335764
MIMAT0009306	TGGTAGACTATGGAACGTAGG	SRR3953059	flex_TRIM_2	132270
MIMAT0009312	ATAGTAGACCGTATAGCGTACG	SRR3953057	RAW	93441
MIMAT0009312	ATAGTAGACCGTATAGCGTACG	SRR3953057	TRIM_1	80924
MIMAT0009312	ATAGTAGACCGTATAGCGTACG	SRR3953057	TRIM_2	11858
MIMAT0009312	ATAGTAGACCGTATAGCGTACG	SRR3953057	flex_TRIM_1	93352
MIMAT0009312	ATAGTAGACCGTATAGCGTACG	SRR3953057	flex_TRIM_2	12643
MIMAT0009312	ATAGTAGACCGTATAGCGTACG	SRR3953058	RAW	86969
MIMAT0009312	ATAGTAGACCGTATAGCGTACG	SRR3953058	TRIM_1	74052
MIMAT0009312	ATAGTAGACCGTATAGCGTACG	SRR3953058	TRIM_2	12358

Continued on next page

Table S6 – *Continued from previous page*

Accession	Sequence	SRA run	type	count
MIMAT0009312	ATAGTAGACCGTATAGCGTACG	SRR3953058	flex_TRIM_1	86892
MIMAT0009312	ATAGTAGACCGTATAGCGTACG	SRR3953058	flex_TRIM_2	13015
MIMAT0009312	ATAGTAGACCGTATAGCGTACG	SRR3953059	RAW	116419
MIMAT0009312	ATAGTAGACCGTATAGCGTACG	SRR3953059	TRIM_1	103729
MIMAT0009312	ATAGTAGACCGTATAGCGTACG	SRR3953059	TRIM_2	12157
MIMAT0009312	ATAGTAGACCGTATAGCGTACG	SRR3953059	flex_TRIM_1	116319
MIMAT0009312	ATAGTAGACCGTATAGCGTACG	SRR3953059	flex_TRIM_2	12830
MIMAT0009340	AGACCCTGGTCTGCACTCTGTC	SRR3953057	RAW	456517
MIMAT0009340	AGACCCTGGTCTGCACTCTGTC	SRR3953057	TRIM_1	189506
MIMAT0009340	AGACCCTGGTCTGCACTCTGTC	SRR3953057	TRIM_2	252307
MIMAT0009340	AGACCCTGGTCTGCACTCTGTC	SRR3953057	flex_TRIM_1	456480
MIMAT0009340	AGACCCTGGTCTGCACTCTGTC	SRR3953057	flex_TRIM_2	267497
MIMAT0009340	AGACCCTGGTCTGCACTCTGTC	SRR3953058	RAW	492330
MIMAT0009340	AGACCCTGGTCTGCACTCTGTC	SRR3953058	TRIM_1	204206
MIMAT0009340	AGACCCTGGTCTGCACTCTGTC	SRR3953058	TRIM_2	272875
MIMAT0009340	AGACCCTGGTCTGCACTCTGTC	SRR3953058	flex_TRIM_1	492279
MIMAT0009340	AGACCCTGGTCTGCACTCTGTC	SRR3953058	flex_TRIM_2	288675
MIMAT0009340	AGACCCTGGTCTGCACTCTGTC	SRR3953059	RAW	574943
MIMAT0009340	AGACCCTGGTCTGCACTCTGTC	SRR3953059	TRIM_1	255838
MIMAT0009340	AGACCCTGGTCTGCACTCTGTC	SRR3953059	TRIM_2	303145
MIMAT0009340	AGACCCTGGTCTGCACTCTGTC	SRR3953059	flex_TRIM_1	574899
MIMAT0009340	AGACCCTGGTCTGCACTCTGTC	SRR3953059	flex_TRIM_2	319765
MIMAT0003843	TGGAAGACTAGTGATTTTGTTGTT	SRR3953057	RAW	1352957
MIMAT0003843	TGGAAGACTAGTGATTTTGTTGTT	SRR3953057	TRIM_1	1258553
MIMAT0003843	TGGAAGACTAGTGATTTTGTTGTT	SRR3953057	TRIM_2	60326
MIMAT0003843	TGGAAGACTAGTGATTTTGTTGTT	SRR3953057	flex_TRIM_1	1352396
MIMAT0003843	TGGAAGACTAGTGATTTTGTTGTT	SRR3953057	flex_TRIM_2	113117
MIMAT0003843	TGGAAGACTAGTGATTTTGTTGTT	SRR3953058	RAW	1654366
MIMAT0003843	TGGAAGACTAGTGATTTTGTTGTT	SRR3953058	TRIM_1	1543477
MIMAT0003843	TGGAAGACTAGTGATTTTGTTGTT	SRR3953058	TRIM_2	73464
MIMAT0003843	TGGAAGACTAGTGATTTTGTTGTT	SRR3953058	flex_TRIM_1	1653492
MIMAT0003843	TGGAAGACTAGTGATTTTGTTGTT	SRR3953058	flex_TRIM_2	132959
MIMAT0003843	TGGAAGACTAGTGATTTTGTTGTT	SRR3953059	RAW	2306690
MIMAT0003843	TGGAAGACTAGTGATTTTGTTGTT	SRR3953059	TRIM_1	2167483
MIMAT0003843	TGGAAGACTAGTGATTTTGTTGTT	SRR3953059	TRIM_2	90324
MIMAT0003843	TGGAAGACTAGTGATTTTGTTGTT	SRR3953059	flex_TRIM_1	2305722
MIMAT0003843	TGGAAGACTAGTGATTTTGTTGTT	SRR3953059	flex_TRIM_2	175801

Table S7. Datasets used in this analysis. Including their accession number, library preparation kit, sample information and reference.

Accession number	Kit	Sample note	Reference
SRR6464623	NEBNext	NN_synth_equi_rep1	Dard-Dascot et al.[1]
SRR6464616	NEBNext	NN_synth_equi_rep2	Dard-Dascot et al.[1]
SRR6464674	CATS	C_synth_equi_rep1	Dard-Dascot et al.[1]
SRR6464673	CATS	C_synth_equi_rep2	Dard-Dascot et al.[1]
SRR6464721	SMARTer	S_synth_equi_rep1	Dard-Dascot et al.[1]
SRR6464720	SMARTer	S_synth_equi_rep2	Dard-Dascot et al.[1]
SRR6464661	TruSeq	TS1_synth_equi_rep1	Dard-Dascot et al.[1]
SRR6464662	TruSeq	TS1_synth_equi_rep2	Dard-Dascot et al.[1]
SRR6464672	NEXTflex	Nf1_synth_equi_rep1	Dard-Dascot et al.[1]
SRR6464671	NEXTflex	Nf1_synth_equi_rep2	Dard-Dascot et al.[1]
SRR3953059	NEBNext	*Bos taurus*	Gümürdü et al.[2]
SRR3953058	NEBNext	*Bos taurus*	Gümürdü et al.[2]
SRR3953057	NEBNext	*Bos taurus*	Gümürdü et al.[2]

Table S8. Trimming sets generated in the analyses with different adapter sequence and corresponding trimming commands.

Trimming set	Description	Execution command
NEBNext_trim01	Trimming with the correct NEBNext adapter sequence 'AGATCGGAAGAGCACACGTCT'	cutadapt -a AGATCGGAAGAGCACACGTCT -o <OUT> <IN>
NEBNext_trim02	Trimming with an additional A at the 5′ end of the NEBNext adapter	cutadapt -a AAGATCGGAAGAGCACACGTCT -o <OUT> <IN>
NEBNext_trim03	Trimming with the incorrect but highly similar CATS adapter 'GATCGGAAGAGCACACGTCTG'	cutadapt -a GATCGGAAGAGCACACGTCTG -o <OUT> <IN>
CATS_trim01	Correct trimming with CATS adapter sequence 'GATCGGAAGAGCACACGTCTG'	cutadapt -a GATCGGAAGAGCACACGTCTG -o <OUT> <IN>
CATS_trim02	Trimming with incorrect but highly similar NEBNext adapter sequence 'AGATCGGAAGAGCACACGTCT'	cutadapt -a AGATCGGAAGAGCACACGTCT -o <OUT> <IN>
CATS_trim03	CATS specific trimming — version January2017	cutadapt -u 3 <IN> | cutadapt -a AAAAAAAA - | cutadapt -a AAAAAAAN$-aAAAAAAN$ -a AAAAAN$ - | cutadapt -a AGAGCACACGTCTG - | cutadapt -O 8 -g GTTCAGAGTTCTACAGTCCGACGATCNNN - | cutadapt -m 18 -o <OUT> -
CATS_trim04	CATS specific trimming — version March2017	cutadapt -u 3 <IN> | cutadapt -a AAAAAAAA - | cutadapt -a AAAAAAAN$ -a AAAAAAN$ -a AAAAAN$ - | cutadapt -a AGAGCACACGTCTG - | cutadapt -O 8 -g GTTCAGAGTTCTACAGTCCGACGATCNNN - | cutadapt -m 18 -o <OUT> -
CATS_trim05	CATS specific trimming — version September2017	cutadapt –trim-n -a GATCGGAAGAGCACACGTCTG -a AGAGCACACGTCTG <IN> | cutadapt -u 3 -a A100 –no-indels -e 0.16666666666666666 - | cutadapt -O 8 –match-read-wildcards -g GTTCAGAGTTCTACAGTCCGACGATCSSS -m 18 -o <OUT> -
CATS_trim06	CATS specific trimming with additional A — version September2017	cutadapt –trim-n -a AGATCGGAAGAGCACACGTCTG -a AGAGCACACGTCTG <IN> | cutadapt -u 3 -a A100 –no-indels -e 0.16666666666666666 - | cutadapt -O 8 –match-read-wildcards -g GTTCAGAGTTCTACAGTCCGACGATCSSS -m 18 -o <OUT> -

<IN>: input raw fastq file. <OUT>: output trimmed file.

References

1. Dard-Dascot, C.; Naquin, D.; d'Aubenton Carafa, Y.; Alix, K.; Thermes, C.; van Dijk, E. Systematic comparison of small RNA library preparation protocols for next-generation sequencing. *BMC Genomics* **2018**, *19*, 118. doi:https://doi.org/10.1186/s12864-018-4491-6.
2. Gümürdü, A.; Yildiz, R.; Eren, E.; Karakülah, G.; Ünver, T.; Genç, Ş.; Park, Y. MicroRNA exocytosis by large dense-core vesicle fusion. *Sci. Rep.* **2017**, *7*, 45661. doi:https://doi.org/10.1038/srep45661.
